# An Uncommon Case of Prosthetic Elbow Joint Infection Caused by Staphylococcus intermedius

**DOI:** 10.7759/cureus.41575

**Published:** 2023-07-08

**Authors:** Zulmarie Rodriguez-Rijos, Alaina S Ritter

**Affiliations:** 1 Division of Infectious Diseases and Global Medicine, University of Florida College of Medicine, Gainesville, USA

**Keywords:** musculoskeletal infection, total elbow replacement, staphylococcus sp, peri-prosthetic joint infection, staphylococcus intermedius

## Abstract

*Staphylococcus intermedius* is known to cause a wide variety of infectious processes in animals and is well described in the veterinary literature. However, the incidence of human infections from this organism has increased in recent years, which highlights the zoonotic potential of this pathogen. Here, we present a case of a *S. intermedius* prosthetic joint infection potentially resulting from exposure to a pet dog.

## Introduction

*Staphylococcus intermedius* is a gram-positive bacterium belonging to the* Staphylococcus* genus. The *Staphylococcus intermedius* group (SIG) includes *S. intermedius*, *S. pseudintermedius*, and *S. delphini* [1,2]. SIG isolates are primarily veterinary pathogens and cause localized and disseminated infections in a wide variety of animal species. However, there has been a recent increase in the number of reported human infections from *S. intermedius* [3]. We describe an uncommon case of a *S. intermedius* prosthetic joint infection (PJI) involving a total elbow joint replacement. To the best of our knowledge, this is only the second reported human case of a PJI associated with *S. intermedius *[3].

## Case presentation

A 79-year-old woman with a past medical history of hypertension, hyperlipidemia, osteopenia, and a right total elbow arthroplasty secondary to inflammatory arthritis presented to our institution with right elbow pain, swelling, and drainage from her prior surgical incision. To summarize her history, she underwent her original right total elbow arthroplasty approximately 15 years prior to presentation due to significant bone-on-bone destruction of her right elbow. She had a negative work-up for rheumatoid arthritis at that time. She did well for approximately six years, at which point she developed increasing elbow pain and limited mobility. She was found to have mild osteolysis around her components and bushing wear. A revision total elbow arthroplasty was performed. 

Seven years later, she again presented with increasing right elbow pain and was found to have loosening of the ulnar component and extensive metallosis. She required a second revision total elbow arthroplasty with tibial shaft allograft for the ulnar component. Intraoperative pathology returned negative for acute inflammation. All surgical cultures were negative. She was noted to have elbow swelling four months postoperatively, but this was diagnosed as bursitis caused by irritation from the indwelling hardware. Her swelling persisted for the next two years with mild pain noted over the ulnar component. The patient then suffered a fall resulting in a lateral ulnar wall fracture with associated pain, erythema, and increased swelling. This was managed non-operatively, but over the next month, she developed drainage from her prior incision. Given concern for active infection, she was placed on empiric oral doxycycline 100 mg twice daily through the orthopedic clinic, which she took for approximately two months in an attempt to avoid surgery. She also received a short course of oral trimethoprim-sulfamethoxazole without improvement. 

**Figure 1:**
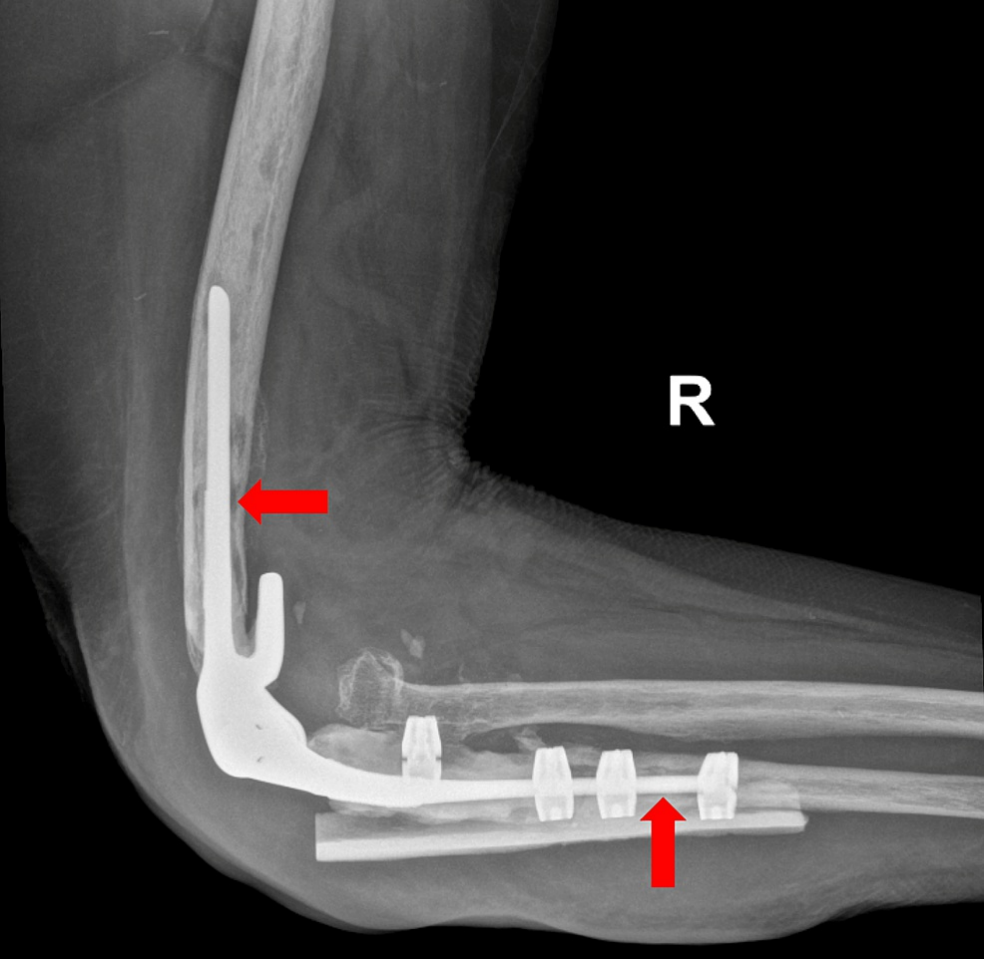
Right elbow X-ray prior to surgery for joint infection Right total elbow arthroplasty with supplemental proximal ulnar diaphyseal onlay allograft. Four radiolucent cerclage devices are in place. There is heterogeneous lucency (red arrows) along the bone-cement interface of the humeral and ulnar stems concerning for loosening without frank changes in alignments. Soft tissue swelling is present around the elbow and proximal forearm. Bony mineralization is decreased diffusely. There is also a chronic deformity of the remnant proximal radius.

The patient ultimately developed increasing drainage, chills, and fatigue and so was admitted to our institution. At presentation, she was afebrile with a temperature of 36.6°C. Blood pressure was 164/80 mm Hg, pulse 74 beats per minute, respiratory rate 14 breaths per minute, and oxygen saturation was 99% on ambient air. Labs were notable for an elevated white blood cell count (12 x 10^3^ cells/mm^3^, normal range: 4.0-10.0) and elevated inflammatory markers with an erythrocyte sedimentation rate of 23 mm/hr (normal range: 0.0-20) and a high-sensitivity C-reactive protein of 24.2 mg/L (normal range: 0.0-4.9). She had a low hemoglobin (11.1 g/dL, normal range: 13.0-16.5) and normal serum creatinine (0.72 mg/dL, normal range: 0.38-1.02). Preoperative X-rays revealed heterogeneous lucency along the bone-cement interface of the humeral and ulnar stems concerning for loosening. There was also soft tissue swelling noted around the elbow and proximal forearm. Bony mineralization was decreased diffusely (Figure 1).

The patient underwent removal of elbow hardware and allograft with debridement of the joint and thorough irrigation. The most proximal cerclage cable could not be retrieved, however, and was left in place. Purulent fluid was noted intra-operatively. A coagulase-positive *Staphylococcus *was identified in five separate cultures encompassing the bone, tissue, and hardware. This organism grew on blood agar after three days of incubation at 37°C with 5% carbon dioxide (CO_2_). Speciation was determined using mass spectrometry (matrix-assisted laser desorption ionization time-of-flight mass spectrometry (MALDI-TOF-MS); bioMérieux Vitek Mass Spectrometry, Nürtingen, Germany) with an adjunctive Remel PYR (L-pyrrolidonyl-beta-naphthylamide) Disk test (Thermo Fisher Scientific, Kansas, USA). The organism was ultimately confirmed as *S. intermedius*; organism susceptibilities are shown in Table 1.

**Table 1:** Susceptibilities of Staphylococcus intermedius

Antibiotic	MIC	Susceptibility
Ciprofloxacin	<=0.5	Susceptible
Daptomycin	0.25	Susceptible
Erythromycin	<=0.25	Susceptible
Gentamicin	>=16	Resistant
Levofloxacin	0.5	Susceptible
Linezolid	1	Susceptible
Minocycline	4	Susceptible
Moxifloxacin	<=0.25	Susceptible
Oxacillin	<=0.25	Susceptible
Penicillin	>=0.5	Resistant
Rifampin	<=0.5	Susceptible
Tetracycline	>=16	Resistant
Trimethoprim-sulfamethoxazole	<=10	Susceptible
Vancomycin	<=0.5	Susceptible

Given the presence of *S. intermedius* in surgical cultures, additional exposure history was obtained from the patient. She denied any recent dental concerns or procedures. She reported having a dog as a pet but did not recall her dog previously coming into close contact with or licking her surgical incision. She noted that her dog was elderly with chronic medical conditions but had not experienced any acute illnesses recently. With the patient’s permission, our team reached out to the veterinarian caring for the dog, who confirmed that the dog had no prior symptoms consistent with *S. intermedius* or another infectious etiology.

Because of limitations related to antibiotic cost, the patient was started on intravenous vancomycin 500 mg every 12 hours with plans to complete a six-week course for PJI. A three-month course of oral rifampin 300 mg twice daily was added for combination therapy given retained hardware in the setting of staphylococcal infection. Once her course of vancomycin was completed, she was transitioned to oral cefadroxil 500 mg twice daily for suppressive therapy. After a risk-benefit discussion with the patient regarding the duration of her cefadroxil suppressive therapy, she elected to continue therapy indefinitely given that she was tolerating it well and wished to minimize the risk of her needing any future surgeries. The patient has continued to follow in the Orthopedics and Infectious Diseases clinics and was doing well one year postoperatively.

## Discussion

The isolates that comprise the SIG have different host associations, but their similar genetic, biochemical, and phenotypic characteristics have historically made it challenging to differentiate them using traditional laboratory methods [1-3]. As a result, some older reports, especially those in dogs and cats, may have actually misidentified *S. pseudintermedius* infections as *S. intermedius* infections [4]. 

*S. intermedius* is a facultative anaerobe and is catalase-positive and coagulase-positive. It is typically a veterinary pathogen that primarily affects dogs and cats, as well as pigeons [1,4]. *S. pseudintermedius* is also a significant pathogen in veterinary medicine and is often associated with canine pyoderma and otitis externa. Like *S. intermedius*, it is both catalase-positive and coagulase-positive [1,5]. By contrast, *S. delphini* is primarily associated with marine mammals, specifically dolphins, and can cause skin lesions and respiratory tract infections. It is catalase-positive but coagulase-negative, distinguishing it from the other two species [1,4]. These differences in host association and coagulase characteristics; the use of phenotypic methods, such as selective culture media, biochemical tests, and antibiotic susceptibility patterns; and the use of advanced molecular techniques, such as real-time polymerase chain reaction (PCR), mass spectrometry (MALDI-TOF-MS), and conventional PCR assays, can assist with the differentiation and classification of these *Staphylococcus *species within the genus. Whole-genome sequencing has also greatly improved our understanding of the genetic diversity within the SIG [4,6,7].

*S. intermedius* is a part of the normal flora in animals and can cause a variety of veterinary infections. These include skin and soft tissue infections, such as dermatitis, pustules, and abscesses, as well as otitis externa, which may result in ear inflammation, pain, and discharge. Urinary tract infections and respiratory tract infections, such as bronchitis or pneumonia, may also occur [8,9]. Human infections with *S. intermedius* are uncommon compared to infections caused by other *Staphylococcus spp.*, such as *S. aureus*. There is a growing body of literature, however, highlighting the occurrence of invasive infections in humans caused by *S. intermedius* [3,10]. These infections, while infrequent, include prosthetic joint infection, skin and musculoskeletal abscesses, bacteremia, infected animal bite wounds, pneumonia, sinusitis, otitis externa, mastoiditis, brain abscesses, and even a case of food intoxication that affected more than 265 individuals in the US in 1991 [3,10-16]. These documented cases, as well as this report, demonstrate the potential for *S. intermedius* to cause significant health problems in humans.

Identified risk factors for *S. intermedius* infections in humans include close contact with animals, particularly dogs, which is significant for individuals who are pet owners or who work in veterinary settings. Dog bites and scratches are also associated with *S. intermedius* infections, particularly skin and soft tissue infections [3,17]. Individuals with compromised immune systems or underlying chronic health conditions, such as diabetes, cancer, immunosuppressive therapy, or indwelling medical devices, may be more susceptible to these infections. The emergence of antibiotic-resistant strains of *S. intermedius* may also pose an increased risk of severe infection [17]. While our patient’s pet dog was the most likely source of her infection, we were unable to confirm that her dog was colonized or infected with the same organism that grew from her operative cultures.

Antibiotics play a crucial role in the treatment of *S. intermedius* infections. The choice of antibiotic depends on the results of susceptibility testing. Oral and IV beta-lactam antibiotics, vancomycin, fluoroquinolones, and adjunctive rifampin are commonly used [3]. Of note, members of the SIG can acquire methicillin resistance through the mecA gene, and an increase in the incidence of resistant isolates has been noted in veterinary specimens [18]. A 2018 study analyzed the susceptibility patterns of 81 SIG isolates from 62 human patients in comparison with 2,655 *S. aureus* isolates obtained from patients during the same time period. Methicillin resistance was noted in 15% of SIG isolates. In addition, SIG isolates were found to be less susceptible to doxycycline (74% susceptible versus 97%) and trimethoprim-sulfamethoxazole (65% susceptible versus 97%) in comparison with *S. aureus* isolates [17]. In our case, the *S. intermedius* isolate was tetracycline-resistant but methicillin-susceptible, and our patient responded well to tailored antimicrobial therapy.

## Conclusions

While the majority of *S. intermedius* infections occur in animals, this case illustrates that it is essential to consider the zoonotic potential of this bacterium. Individuals who have close contact with potentially infected or colonized animals may be at an increased risk of infections. Prompt diagnosis and appropriate treatments are important to ensure the effective management of *S. intermedius*-associated infections in a clinical setting.
